# Efficacy of a county-wide schools weight management intervention

**DOI:** 10.1177/17579139211006738

**Published:** 2021-05-15

**Authors:** GJ Sanders, WL Marwa, B Wade, P Gately

**Affiliations:** Carnegie School of Sport, Leeds Beckett University, Fairfax Hall Rm 230, Headingley Campus, Leeds LS6 3QS, UK; Carnegie School of Sport, Leeds Beckett University, Leeds, UK; OneLife Suffolk, Inspire Suffolk, Ipswich, UK; Carnegie School of Sport, Leeds Beckett University, Leeds, UK

**Keywords:** childhood, obesity, prevention, intervention, weight management

## Abstract

**Aims:**

This study aimed to evaluate the effectiveness of the Local Authority commissioned large-scale public health service that provided a 6-week school-based weight management intervention for children aged 4–19 years.

**Methods:**

A quantitative retrospective cohort study identified participants from 130 schools consisting of 8550 potential children aged 4−19 years across a mixture of Lower Super Output Area (LSOA) deprivation groups. Participants were invited to take part in a 5- to 12-week Healthy Lifestyles intervention with a focus on weight management delivered by OneLife Suffolk between 1 January 2017 and 1 January 2020. This resulted in a final sample of 5163 participants. The following information for each child was collected anonymously: (1) age, (2) gender, (3) preprogramme body mass index (BMI), (4) postprogramme BMI, (5) weight category and (6) LSOA category.

**Results:**

Following the 6-week school-based intervention, there was a significant decrease in mean ΔBMI SDS (standardised body mass index) of −0.07 (−14.89%) among participants. Wilcoxon signed-rank test showed a significant change in weight status post 6-week weight management programme (WMP): BMI (*Z* = −15.87, *p* < .001), BMI SDS (*Z* = −21.54, *p* < .001), centile (*Z* = −20.12, *p* < .01) and weight category (*Z* = −7.89, *p* < .001), whereas Mann−Whitney *U* test showed no statistically significant difference in mean BMI SDS change between gender groups (*p* = .24) and Kruskal−Wallis test revealed no statistically significant differences in mean BMI SDS change between child LSOA groups (c^2^(4) = 1.67, *p* = .796), school LSOA groups (c^2^(4) = 4.72, *p* = .317), ethnic groups (c^2^(4) = 2.53, *p* = .640) and weight category at the start of the intervention (c^2^(3) = 6.20, *p* = .102).

**Conclusions:**

This study contributes to the growing body of evidence demonstrating the efficacy of multicomponent school-based weight management interventions and demonstrates that such interventions can be successfully implemented as part of a wider healthy lifestyles service, without widening health inequalities.

## Introduction

The global prevalence of childhood obesity has increased more than eightfold among 5- to 19-year-olds over the past four decades, and continues to rise.^1^ Although the increase in mean body mass index (BMI) is consistent on a global scale, obesity prevalence has accelerated in east and south Asia for both sexes, and southeast Asia for boys.^1^ Hence, promoting the health of disadvantaged children, both in low- and low-medium-income countries and in disadvantaged groups in affluent countries, requires particular attention.

In England, 22.4% of reception-aged children suffer from overweight or obesity, rising to 34.3% for children aged 10−11 years and 40% for children aged 13−15 years.^2^ Alarmingly, severe obesity among this age group continues to rise and has increased by more than a third since 2007 to 4.2%, the highest rate recorded to date.^2^ Severe childhood obesity remains a growing yet under-recognised health problem.

Children who suffer from overweight and obesity are more susceptible to developing both physical (e.g. type II diabetes, musculoskeletal disorders and respiratory problems) and psychosocial (e.g. self-esteem, quality of life, stigmatisation and depression) issues.^3,4^ When compared with children suffering from moderate obesity, children suffering from severe obesity are at an even greater risk of such health problems.^5^ The model for mediating (i.e. factors which help explain the relationship between two conditions) and moderating (i.e. factors that might influence the strength of a relationship between two conditions) factors^6^ shows that the relationship between childhood obesity and physical and psychosocial health is bidirectional. Moderating factors in children include the following: boys, older children (13−15 years), of a lower socioeconomic status (SES), disabled and of a Black ethnicity. Mediating factors include behavioural (e.g. diet and exercise adherence), biological (e.g. chronic disease and medication use), psychological (e.g. poorer perceived health, negative thoughts and low self-esteem) and social factors (e.g. stigmatisation and low social support).

Of particular importance in the UK is the influence of SES. In the most deprived areas in England, 12.8% of children in age 4−5 years suffer from obesity compared with 5.7% in the least deprived. Among children aged 10−11 years, this percentage is 26.8% in the most deprived areas, compared with 11.7% in the least deprived.^1^ Furthermore, significantly higher levels of severe obesity have been reported in areas of low SES.^5^ Families from low-income communities are faced with several potential barriers to preventing improvement in health statuses: access to physical activity (PA) opportunities, neighbourhood safety, cost, transport, and knowledge and education of healthy behaviours.^7,8^ Furthermore, families with low SES are less likely to recognise a child as being in the overweight or obese categories^1^ and thus do not believe that an intervention is required to change a child’s eating and activity behaviours.^9^ Recognising signs of childhood obesity is a key challenge to reduce further enhancing health inequalities, and hence, education for children and parents is key.^10^

Marmot^11^ describes a gradient of inequity in health risks across the population and advises proportionate universalism to tackle this. In other words, that more effort be put into assisting those who are considered the most vulnerable (e.g. moderating risk factors). Childhood weight management interventions should strive for suitability and effectiveness across a universal spectrum of participant characteristics in order to decrease attrition as change in standardised body mass index (BMI SDS) is positively correlated to programme completion rates.^12^ Despite this, such services are only available to a small number of those in need across England.^13^

A large amount of a child’s time between the ages of 4 and 16 years is spent within a school environment. Between January 2017 and January 2018, the number of pupils enrolled in school in England was 8,735,098.^14^ This offers an opportunity to use policies, staff, curricula and parental engagement to positively influence a child’s health and wellbeing. Given the wide reach of schools and the fact that they provide a platform for equity and a relative consistency of information translation, they present an opportunity to address obesity without widening health inequalities further.^15^ Despite this, evidence of weight management intervention impact in schools is mixed. A recent systematic review and meta-analysis of the overall effects of 50 randomised controlled trial (RCT) school-based obesity prevention interventions showed that short-term (6- to 12-week) interventions are more effective in reducing weight among overweight and obese children than long-term (>12-week) interventions.^15^ Concurrently, recent evidence of the large-scale (*n* = 1467 pupils) 12-month West Midlands ActiVe lifestyle and healthy Eating in School children (WAVES) intervention^16^ concluded no evidence of clinical effectiveness or cost-effectiveness. A lack of knowledge, awareness and skills to deal with the sensitivity and complexity of childhood obesity across all school stakeholders presents the most significant barrier to effective action.^17^

There is a recognition in the literature that obesity is a complex issue requiring system-based approaches (i.e. individually tailored approaches informed by theory about complex systems which propose new ways of organising, managing and evaluating activities).^18^ Given findings that long-term RCT interventions may result in decreased child enjoyment, motivation and subsequent retention,^19^ short-term (6- to 12-week interventions), pragmatic school-based weight management interventions, using some elements of systems thinking, could provide a more cost-effective way to evaluate effectiveness and subsequently test and modify through ‘trial and error’ intervention components within ‘real world’ settings.^20^ Pragmatic interventions within ‘real world’ settings enable mutual learning and understanding about the activities, opinions, values and experiences of not only participants themselves, but also of organisational structures and diverse stakeholder groups (e.g. parents and teachers). This approach enables efficacious pilot interventions to be ‘scaled-up’ into county-wide trials across local authorities. This is in line with the physical and health education (PHE) guide to supporting local approaches in promoting a healthy weight.^21^ The available global evidence indicates large benefits of promoting healthy eating patterns and limiting sugar-containing beverage consumption from early childhood onwards.^22^ Regular PA and limited sedentary lifestyle and screen time alone have limited effects but are valuable elements in effective multicomponent strategies.^22^

Therefore, this study explored the impact of a pragmatic 6-week school-based local authority supported intervention on a large number of schools across an English rural county. Our secondary aim was to determine intervention impact on a number of variables (i.e. age, gender, ethnicity and SES) that are associated with health inequalities.

## Methods

This study provides quantitative data within a community-based Integrated Healthy Lifestyle Service (IHLS). The observed IHLS focuses on reducing health inequalities among vulnerable and hard-to-reach groups within areas of deprivation. The service is a partnership between a UK-based university and is commissioned by a local County Council in the south east of England.

### Participants and procedures

**Figure 1:**
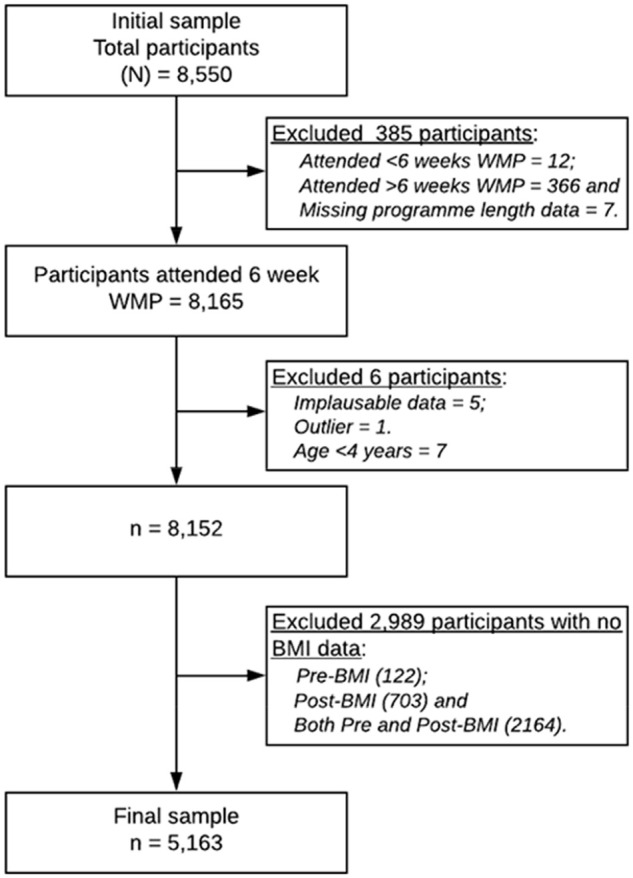
Flowchart of data management WMP: weight management programme; BMI: body mass index.

A quantitative retrospective cohort study was used to generate relevant data for this study. This study involved 8550 children from different socioeconomic backgrounds, aged 4−19 years from 130 schools attending a 5- to 12-week weight management programme (WMP). Of all participants, 8165 participants attended a 6-week WMP. After excluding participants with implausible data, outliers and missing BMI data, the final sample remaining was 5163 (Figure 1). All the participants involved in the analysis completed the 6-week WMP, between 1 January 2017 and 1 January 2020, delivered by OneLife Suffolk. Details of the flow of participants through the study from baseline to follow-up are displayed in Figure 1.

Ethical approval was provided by Leeds Beckett University’s (LBU) Research Ethics Sub-Committee (Approval No. 72597). Secondary data sampling was implemented via the primary data source of the public health initiative OneLife Suffolk, to generate participants, that had provided parental consent for children who had completed the 6-week school-based weight management intervention. The study solely focussed on participants fitting the above criteria in interventions delivered in the county of Suffolk. To be eligible for inclusion, participants had to be aged between 4 and 19 years and completed OneLife Suffolk’s 6-week school-based weight management intervention. Participation was voluntary with no incentives provided.

### Intervention

The OneLife Suffolk Healthy Schools Programme is part of a wider healthy lifestyles service that is funded by the Public Health department at Suffolk County Council. The intervention is an evidence-based, multicomponent school-based WMP, developed by a specialist team of clinicians, including a dietician and health psychologist, with a strong knowledge of obesity. The programme follows the Standard Evaluation Framework (SEF) for Weight Management Interventions good practice in behaviour change guidelines.^23^

The intervention is delivered by trained OneLife practitioners and consists of six healthy lifestyle workshop style sessions (see Table 1). Sessions provide evidence-based, public health messaging around the key topics designed to support lifetime healthy lifestyle skills and knowledge to promote and encourage long-term maintenance of healthy eating choices and increased PA. Parents also receive healthy lifestyle parent manuals, and optional training specific to school staff and their role in children’s health is provided where warranted. The programme has three different curriculums to ensure age-appropriate strategies are delivered to children from reception age (4−5 years old) through to year 12 (18 years old). The skeleton curriculum has been developed in line with the SEF for Weight Management Interventions’ good practice in behaviour change guidelines.^24^ however, the intervention further extends this by introducing the four key constituents of the self-theory,^25^ which include self-awareness, self-regulation, self and others, and self-reliance. It also uses self-determination theory^26^ which supports behaviour change by promoting competence (knowledge and skills of eating and activity behaviours), autonomy (planning, goal setting, monitoring) and relatedness (through the inclusion of peers, teachers and parents to achieve common goals).

**Table 1:** Example content from the OneLife Suffolk Healthy Schools Programme

Study week	Healthy lifestyle messages
Week 1	Healthy lifestyle, healthy body
Week 2	Healthy balanced diet and portion sizes
Week 3	Regular eating and healthy snacks
Week 4	Importance of PA and reducing sedentary behaviours
Week 5	Understanding food labels and sugary drinks
Week 6	The importance of sleep

PA: physical activity.

To facilitate successful implementation of the intervention, OneLife practitioners invited school staff to a training session before the intervention began, to provide staff with the aims, objectives and ethos of the intervention. This was to prepare school staff themselves to best help motivate children and parents to take the healthy lifestyle message on board and encourage intervention attendance as well as address potential questions/worries school staff may have. Following full completion of the 6-week intervention, children received a certificate of achievement. Incentives and rewards for continued attendance, including certificates of achievement and progress reports, are noted as being particularly important for children.^26^

### Measures

#### Height and weight

Height and weight measurements were taken preintervention and postintervention, and BMI centile was precalculated using the Microsoft Excel add-in LMSgrowth.^27^ All measurements were carried out by a Healthy Lifestyle Practitioner employed by OneLife Suffolk. Weight measurements were taken in light clothing without shoes using portable digital scales (Seca 875 Flat Scales for Mobile Use) to the nearest 0.1 kg. Height was recorded to the nearest 0.1 cm using a portable stadiometer (Marsden HM-250P Leicester Portable Height Measure). BMI was calculated using the equation weight (kg)/height (m)^2^, and BMI SDS was calculated using the ‘LMS’ method.^28^

#### SES status

Postal codes were used to estimate SES by generating Index of Multiple Deprivation (IMD)^29^ using an online conversion tool (http://imd-by-postcode.opendatacommunities.org/). The IMD is a UK government metric used to rank area-level deprivation within and between different communities. The IMD scores rank each super output area in England from 1 (most deprived area) to 32,844 (least deprived area). The IMD considers seven domains which relate to health deprivation and disability, education skills and training deprivation, income deprivation, employment deprivation, barriers to housing and services, living environment deprivation, and crime.^29^ For the purpose of this study, Lower Super Output Areas (LSOAs) were categorised into five subgroups for analysis: group 1 (lowest deprivation) = 0%−20%, group 2 = 21%−40%, group 3 = 41%−60%, group 4 = 61%−80% and group 5 (highest deprivation) = 81%−100%.

#### Gender and age

Before OneLife Suffolk delivered the 6-week intervention, a form was sent out to the parents for them to complete and sign. This form asked for the child’s date of birth and gender. For anonymity purposes, OneLife Suffolk only shared the age of the child, not the date of birth.

Confidentiality and data storage procedures were adhered to as is set out in the Leeds Beckett Data Management Plan.^30^ All participant data were anonymised and coded to prevent identification, and securely stored using password-protected files on the LBU computing network. Only research team members had access to the anonymised data. This was shared between the team strictly for the purposes of research.

### Data coding and analysis

All data were analysed using IBM SPSS Statistics for Windows version 25.0 (IBM, Armonk, NY, USA). Most of the variables in the data were complete (82.96%); however, some variables had missing values as follows: Child LSOA: 360 (7.0%), Child IMD Rank: 360 (7.0%) and Ethnicity 2656 (51.4%). Multiple imputation was used to optimise power and maintain the sample size by generating several imputed data sets based on the observed data.^31,32^ Independent analyses were conducted on each data set, and then a single estimate was finally generated by pooling results of each imputed data set.^31,32^

Descriptive statistics (e.g. frequency, mean and standard deviation (SD)) were calculated for participants characteristics and other measured variables. To guide a choice of the appropriate test for the analysis, tests of normality were performed. The assumption of normality for BMI SDS change was not satisfied as assessed by Shapiro−Wilk’s test (*p* < .01) and by visual inspection of normal Q−Q plots. Due to violation of normality assumption, non-parametric tests such as Mann−Whitney *U* and Kruskal−Wallis *H* were used to examine mean differences of BMI SDS change between groups in binary variables (e.g. gender (male/female)) and variables with >2 categories (e.g. ethnicity), respectively. Wilcoxon signed-rank test was used to investigate change in BMI, BMI SDS and weight category after 6 weeks of intervention. Furthermore, logistic regression analysis was performed to assess the relationship between BMI SDS loss (outcome variable) and predictor variables (i.e. participants’ sociodemographic characteristics, weight category and BMI SDS at the start of intervention).

## Results

The study involved children aged 4−19 years with mean age of 8.8 ± 2.3 years (Table 1). Around half of participants (50.1%) were males and the other half (49.9%) were females. Each child LSOA category contained roughly a fifth of all participants. Majority of participants (66.3%) were White and the rest were from other ethnic groups. The mean BMI at the start and end of intervention were 17.62 ± 3.04 and 17.52 ± 3.04 kg/m^2^, respectively. Likewise, the mean BMI SDS at the start and end of intervention were 0.47 ± 1.13 and 0.40 ± 1.14, respectively. Majority of participants (65.7%) had BMI SDS maintained or lost, whereas roughly a third (34.3%) of participants gained BMI SDS.

Following the 6-week school-based healthy living programme, there was an observed mean ΔBMI SDS of −0.07 (−14.89%) among participants. Importantly, while there were −9.43% and −6.25% decreases in children in overweight and very overweight categories, respectively, the healthy weight BMI SDS category had a 2.16% increase of children (Table 2).

**Table 2:** Participant characteristics

	*n* (%)
Gender	
Female	2577 (49.9)
Male	2586 (50.1)
Age at the start of intervention (years): Min = 4; max = 19; mean ± SD = 8.8 ± 2.3	
Ethnicity	
White	3430 (66.4)
Black	993 (19.2)
Asian	336 (6.5)
Mixed	82 (1.6)
Any other	328 (6.3)
Child LSOA	
1	990 (19.2)
2	1089 (21.1)
3	1110 (21.5)
4	1073 (20.8)
5	901 (17.5)
School LSOA	
1	1009 (19.5)
2	998 (19.3)
3	1004 (19.4)
4	1401 (27.1)
5	751 (14.5)
BMI SDS maintained/loss	
No	1772 (34.3)
Yes	3391 (65.7)
Category at the start of intervention	
Healthy range	3645 (70.6)
Close to overweight	370 (7.2)
Overweight	349 (6.8)
Very overweight	799 (15.5)
Category at the end of intervention	
Health range	3723 (72.1)
Close to overweight	375 (7.3)
Overweight	316 (6.1)
Very overweight	749 (14.5)

SD: standard deviation; LSOA: Lower Super Output Area; BMI SDS: standardised body mass index.

A Wilcoxon signed-rank test showed that the 6-week school-based healthy living programme resulted in decreased BMI (*Z* = −15.87, *p* < .001), BMI SDS (*Z* = −21.54, *p* < .001), centile (*Z* = −20.12, *p* < .01) and weight category (*Z* = −7.89, *p* < .001). Meanwhile, the Mann−Whitney *U* test showed no statistically significant difference in mean BMI SDS change between males and females (*p* = .24). The Kruskal−Wallis test also revealed no statistically significant differences in mean BMI SDS change between child LSOA groups (c^2^(4) = 1.67, *p* = .796), school LSOA groups (c^2^(4) = 4.72, *p* = .317), ethnic groups (c^2^(4) = 2.53, *p* = .640) and weight category at the start of the intervention (c^2^(3) = 6.20, *p* = .102). This indicates equal effectiveness of the 6-week WMP across different groups of gender, ethnicity, LSOA and weight category at the start of the intervention. Furthermore, logistic regression analysis revealed increased odds of achieving BMI SDS loss by a factor of 1.3 (95% confidence interval (CI) = 1.093−1.464) for each unit increase in child BMI SDS at the start of the intervention. Other individual characteristics at the start of the intervention were not predictive of the BMI SDS loss (Table 3) or change (Table 4).

**Table 3:** Change in BMI, BMI SDS and weight category at the start and end of the intervention

	Min	Max	Mean ± SD	
BMI start	11.98	39.43	17.62 ± 3.04	
BMI end	11.66	39.43	17.52 ± 3.04	
BMI-SDS start	−3.72	4.77	0.47 ± 1.13	
BMI-SDS end	−4.18	4.77	0.40 ± 1.14	
Centile end	0	1	0.6 ± 0.3	
Centile start	0	1	0.6 ± 0.3	
	Start	End	Δ	%Δ
Mean BMI	17.62	17.55	−0.07	−0.40
Mean BMI SDS	0.47	0.40	−0.07	−14.89
Centile	0.614	0.595	−0.019	−3.09
Weight category start of intervention				
Healthy weight	3649	3728	79	2.16
Close to overweight	371	375	4	1.08
Overweight	350	317	−33	−9.43
Very overweight	800	750	−50	−6.25

BMI: body mass index; BMI SDS: standardised body mass index; SD: standard deviation.

**Table 4:** Results of a regression assessing associations between BMI SDS change (outcome variable) and individual characteristics

	β (95% CI)	*p* value
Constant	2.846 (1.039–7.792)	.042
Age start	1.021 (0.984–1.059)	.266
Gender		
Male	0.976 (0.868−1.098)	.692
Ethnicity		
White	1.054 (0.567–1.957)	.862
Black	1.002 (0.386−2.600)	.997
Asian	1.098 (0.461−2.615)	.826
Mixed	1.298 (0.589−2.860)	.509
Child LSOA		
1	0.941 (0.758−1.170)	.583
2	0.961 (0.779−1.186)	.712
3	0.783 (0.642−0.954)	.015
4	0.968 (0.791−1.184)	.749
BMI start	0.965 (0.909−1.025)	.248
BMI SDS start	1.300 (1.093−1.464)	.002*

BMI SDS: standardised body mass index; CI: confidence interval; LSOA: Lower Super Output Area; BMI: body mass index.

Variables entered in the model were gender, age start, ethnicity, child LSOA, BMI start, BMI SDS start.

* Reached statistical significance of *p* < .05.

## Discussion

This is one of the first UK-based studies to examine the effectiveness of the large-scale pragmatic 6-week multicomponent school-based weight management intervention. Results revealed significant BMI and BMI SDS losses and weight category changes following the 6-week weight management intervention regardless of child age, gender, ethnicity, LSOA and weight category at the start of intervention.

An observed mean decrease in BMI SDS of 0.07 was reported in this study. A recent overview of Cochrane reviews among interventions for treating children and adolescents with overweight and obesity reported an overall reduction in BMI SDS of 0.06 among children aged 6−11 years and 0.1 among children ⩾12 years of age.^33^ Furthermore, a recent meta-analysis of school-based weight management interventions outlined that across 50 trials, single-component interventions resulted in a BMI SDS reduction of 0.05, while multicomponent interventions resulted in a BMI SDS reduction of 0.07.^15^ While any reduction in BMI SDS for children with overweight and obesity may be of clinical benefit, the BMI SDS reduction required to ameliorate any comorbidities is less clear. However, improvements in cholesterol were observed in children with obesity aged 7−17 years with a BMI SDS reduction of <0.1 unit,^34^ and improvement in insulin and cholesterol was observed in 5- to 19-year-olds with obesity, following a BMI SDS reduction of 0.15 (SD = 0.5) units.^35^ These findings highlight the potentially beneficial clinical effects of the OneLife Suffolk pragmatic intervention and its appropriateness among children of a wide range of ages, socioeconomic background and initial weight. This is of particular importance as the intervention further demonstrates the potential efficacy of pragmatic short-term (6- to 12-week) interventions,^15^ as well as outlining their potential in reducing the widening of health inequalities among this population.^11^

School settings offer an opportunity to use policies, staff, curricula and parental engagement to positively influence a child’s health and wellbeing.^36^ Taken collectively, the evidence from recent systematic reviews and meta-analyses^15,37^ suggests that multicomponent school-based intervention programmes involving activities to engage children and their parents are most effective in achieving small reductions in body weight status in children of all ages.^15^ However, the review identifies significant between-study heterogeneity and acknowledges that most of the included studies have a moderate-to-high risk of bias. The large sample size and comparable age, gender and LSOA distributions in this study significantly reduced the risk of bias, and thus, results can be considered as representative. Furthermore, theoretically informed interventions have been found feasible and acceptable to schools, children and their families and have achieved the highest levels of engagement.^36^ The OneLife Suffolk curriculum has been developed in line with the SEF for Weight Management Interventions good practice in behaviour change guidelines.^23^ OneLife Suffolk further extends this by introducing the four key constituents of the self-theory,^24^ which include self-awareness, self-regulation, self and others and self-reliance, supported by the use of self-determination theory^25^ to deliver and promote individually tailored sessions (e.g. individualised goals based upon history, goals and ability). This method has shown to have the greatest likelihood of promoting sustainable long-term weight loss.^37^ Specifically, OneLife Suffolk sessions sought to provide children with the necessary skills to identify and make healthy diet and activity choices and engage their parents and peers in supporting these behaviours. Children were given age-appropriate levels of autonomy to select which behaviours they wished to change, and parents were encouraged to identify how they would support their child to achieve their goals.

Previous research shows that childhood obesity management tends to reproduce health inequalities between children.^10^ Specifically, when accessing community-based WMPs, children with a high-SES encounter less difficulties in adapting their lifestyle to professionals’ recommendations than low-SES children because their habitus facilitates the internalisation of health norms and they have greater access to economic, social and cultural capitals.^10^ Consequently, schools are ideal locations for childhood weight management interventions given their near-universal reach of children across the socioeconomic spectrum, and the significant weight loss findings in this study across schools regardless of child age, gender, ethnicity, LSOA and initial weight support this.

Findings should be interpreted in the context. The reporting of intervention characteristics (dose, frequency and content) varied so much between sessions that no specific intervention content could be attributed as either being more or less effective. A 2005 Cochrane systematic review^38^ recommended that interventions designed to prevent childhood obesity should have a rigorous assessment design that enables sufficiently powered analysis of what is working or not and for whom the intervention is working, and that stakeholders should be included in the development of the programme. However, it has been demonstrated that long-term RCT interventions adopting a strict protocol consisting of the same components for all clients regardless of ability may result in decreased child enjoyment, motivation and subsequent retention.^19^ Consequently, pragmatic multicomponent interventions utilising the principles of systems thinking could provide a more cost-effective way to evaluate effectiveness and subsequently test and modify through ‘trial and error’ intervention components within ‘real world’ settings.^20^ The scale of childhood obesity warrants future childhood weight management interventions to explore effectiveness across sectors (e.g. school, community, home-based) and levels (e.g. tier 1, 2, 3 and 4 services), as well as including detailed descriptions of approaches, content and embedded process and economic evaluations, as recommended by existing guidance on developing and evaluating complex interventions.^39^ To achieve this, more qualitative research conducted to understand the barriers and facilitators to child weight management interventions is warranted,^37^ as well as more process evaluations^39^ to help guide implementation and tailor interventions specifically to this populations needs.

Since 2010, local governments in the UK have seen significant reductions in public health resources;^40^ therefore, pragmatic interventions that demonstrate reach and impact are required to enable public health professionals to use financial resources wisely. Community weight loss programmes that specifically target children suffering from weight problems have been shown to be effective, but they can be difficult to run in isolation (which is how they are often commissioned) and recruit, especially in areas where the population is dispersed. Therefore, considerations about the appropriate mix of services, universal and targeted interventions, are warranted and are likely to differ in each area dependent on needs and resources. This intervention demonstrates changes of magnitude (0.07 BMI SDS) similar to those reported in targeted community intervention programmes.^33^ This suggests that those commissioning local services have more than just targeted interventions as part of their local actions, although ideally such intervention options should be led by the needs of children and young people as well as the health and wellbeing strategies of the local public health teams.

Methodological strengths include the large sample size and comparable age, gender and LSOA distributions which ensured results are representative of children aged 4−18 years of age across Suffolk county. In line with the SEF for weight management interventions,^22^ the design, delivery and recruitment strategies were theoretically underpinned by conceptual behaviour change models.^24,25^

Limitations are also noted. The purposeful recruitment process prevents the calculation of a precise response rate and may limit the representativeness of the sample. The large proportion of children and families that did not consent to their data being used is a limitation; this was a pragmatic local intervention and we hope to address this issue in future programmes. Nonetheless, key characteristics of participants in this study such as age, LSOA, BMI and BMI SDS were very similar to those of previous school-based weight management interventions.^15^ Furthermore, the cross-sectional design of the study ensures that the findings represent associations between BMI and the other variables, rather than imply a causal relationship.

## Conclusion

This study contributes to the growing body of evidence demonstrating the efficacy of short-term, pragmatic multicomponent school-based weight management interventions and is a first step in demonstrating an increased understanding of systems thinking that may result in weight loss among children in the UK. Findings suggest that a 6-week multicomponent school-based weight loss intervention can be effective regardless of child age, gender, ethnicity, LSOA and weight category at the start of intervention. The accumulating evidence may also help inform national-level policy and intervention strategies aimed at reducing childhood obesity.
